# A Comparative Study on Microstructure, Segregation, and Mechanical Properties of Al-Si-Mg Alloy Parts Processed by GISS-HPDC and SEED-HPDC

**DOI:** 10.3390/ma16206652

**Published:** 2023-10-11

**Authors:** Guo-Chao Gu, Li-Xin Xiang, Rui-Fen Li, Wen-Hua Xu, Hong-Liang Zheng, Wen-Hao Wang, Yu-Peng Lu

**Affiliations:** 1 Key Laboratory for Liquid-Solid Structural Evolution and Processing of Materials, Ministry of Education, Shandong University, Jinan 250061, China; 202034221@mail.sdu.edu.cn (L.-X.X.); xuwh2009@sdu.edu.cn (W.-H.X.); honglz@126.com (H.-L.Z.); 2 School of Materials Science and Engineering, Shandong University, Jinan 250061, China; 3 Suzhou Institute, Shandong University, Suzhou 215123, China; 4 Shandong Institute for Product Quality Inspection, Jinan 250102, China; liruifen2016@163.com; 5 School of Materials Science and Engineering, Shandong Jianzhu University, Jinan 250101, China; a3068880370@163.com

**Keywords:** GISS, SEED, semi-solid die-casting, mechanical properties

## Abstract

There are multiple routes to prepare semi-solid slurries with a globular microstructure for semi-solid forming. The variations in the microstructure of semi-solid slurries prepared using different routes may lead to significant differences in the flow behavior and mechanical properties of rheo-diecasting parts. Therefore, it is crucial to have a comprehensive understanding of the microstructure evolution associated with different slurry preparation routes and their resulting effects. In this study, the gas-induced semi-solid process (GISS) and the swirl enthalpy equilibrium device (SEED) routes were employed to prepare semi-solid Al-Si-Mg slurries for their simplicity and productivity in potential industrial applications. The prepared slurries were then injected into the shoot sleeves of a high-pressure die casting (HPDC) machine to produce tensile test bars. Subsequently, the bars underwent T6 treatment to enhance their mechanical properties. The microstructure, segregation, and mechanical properties of the samples were investigated and compared with those of conventional HPDC. The results indicated that the GISS and SEED can produce semi-solid slurries containing a spherical α-Al primary phase, as opposed to the dendritic structure commonly found in conventional castings. The liquid fraction had a significant effect on the flow behavior, resulting in variations in liquid segregation and mechanical properties. It was observed that a higher solid fraction (>75%) had a suppressing effect on surface liquid segregation. In addition, the tendency for liquid segregation gradually increased along the filling direction due to the special flow behavior of the semi-solid slurry with a low solid fraction. Furthermore, under the same die-casting process parameters, the conventional HPDC samples exhibit higher yield stress (139 ± 3 MPa) compared to SEED-HPDC and GISS-HPDC samples, which may be attributed to the small grain size and the distribution of eutectic phases. After undergoing the T6 treatment, both SEED-HPDC and GISS-HPDC samples showed a significant improvement in yield and tensile strength. These improvements are a result of solution and precipitation strengthening effects as well as the spheroidization of the eutectic Si phase. Moreover, the heat-treated SEED-HPDC samples demonstrate higher ultimate strength (336 ± 5 MPa) and elongation (13.7 ± 0.3%) in comparison to the GISS-HPDC samples (307 ± 4 MPa, 8.8 ± 0.2%) after heat treatment, mainly due to their low porosity density. These findings suggest that both GISS-HPDC and SEED-HPDC processes can be utilized to produce parts with favorable mechanical properties by implementing appropriate heat treatments. However, further investigation is required to control the porosities of GISS-HPDC samples during heat treatment.

## 1. Introduction

High-pressure die-casting (HPDC) technology is a highly productive near-net-shape forming process used for aluminum and magnesium alloys. It has found applications in various industries, such as automotive, aerospace, and 5G communications [1,2]. Blisters may arise during heat treatment due to air entrapment in the die-casting process, rendering the die-casting parts unsuitable for heat treatment [3]. Consequently, the application of die castings with high strength is limited. To overcome this situation, several enhanced casting processes have been developed, such as high vacuum die-casting, semi-solid die-casting (rheo-HPDC), etc. [4,5]. In the case of semi-solid die-casting, the higher viscosity promotes a laminar flow of the slurry in the sleeve and cavity, characterized by a reduced occurrence of issues like oxidation [2], shrinkage [6], and air entrapment [7]. Moreover, compared to conventional HPDC, rheo-HPDC involves keeping the alloy at a semi-solid temperature for a long period of time while ensuring a faster cooling rate during the cooling process. As a result, the constituents become more uniform, and the size of eutectic and intermetallic phases located near the α-Al phase is reduced. These factors can greatly reduce the solution time required for the T6 heat treatment [8].

In the rheo-HPDC process, the solid phase and liquid phase exhibit different flow behavior, which leads to segregation phenomena. Feng [9] utilized the rheo-HPDC technique to produce Al-6Si-3Cu-0.4Mg aluminum alloy castings. It was suggested that the variation in liquid fraction between the edge and center of the samples results in different mechanical properties. Govender [10] investigated the surface segregation phenomenon of A356 aluminum alloy in die-casting and observed variations in the thickness of the segregation layer at different positions of the casting. This variation in thickness was found to enhance the hardness at specific positions, while not significantly affecting the overall mechanical properties. Segregation in the die-casting process can result in an uneven microstructure, which can subsequently affect the mechanical properties of the product. Hence, it is crucial to investigate the segregation behavior during the die-casting process.

One of the key factors affecting rheo-HPDC is the preparation of semi-solid slurries with a uniform and spherical microstructure. Since Flemings obtained a semi-solid slurry with primary spherical grains by stirring Sn-15%Pb alloy in the 1970s, researchers have invented some new methods to prepare semi-solid slurries [11]. Alcan [12] patented the SEED method, which involves preparing a semi-solid slurry through heat exchange and eccentric rotation. Wannasin [13] conducted research on the GISS technology, which enables a uniform distribution of temperature and solute within the melt through argon convection and stirring of the graphite rods. This technique effectively limits the growth of dendrites in primary grains. Lv [14] developed the IUV (Indirect Ultrasonic Vibration) technology, which utilizes the vibratory effect of a horn to produce semi-solid slurries. Zhu [15] developed the SCP (Serpentine Pouring Channel) technology, where the stirring effect of the serpentine channel gradually transforms primary grains into nearly spherical grains. Jain [16] investigated the RSF (Rapid Slurry Formation) technology, which utilizes an enthalpy exchange material (EEM) for slurry preparation.

Although many researchers have studied the application of GISS and SEED in different alloys and demonstrated their potential for industrialization, there are fewer studies on the two processes in relation to the forming and mechanical properties of the same alloy. Therefore, the aim of this study is to comparatively investigate the microstructure, flow behavior, and mechanical properties of the alloy produced by different rheo-HPDC processes. In this study, the semi-solid Al-Si-Mg alloy slurries were prepared by the GISS and SEED routes. The prepared slurries were then injected into the shoot sleeves of a high-pressure die casting (HPDC) machine to produce tensile test bars. Subsequently, the bars underwent T6 treatment to enhance their mechanical properties. The differences in the slurry microstructure of the two were examined. The segregation and mechanical properties of the samples produced by GISS-HPDC and SEED-HPDC were compared and analyzed. 

## 2. Materials and Methods

### 2.1. Materials

**Figure 1:**
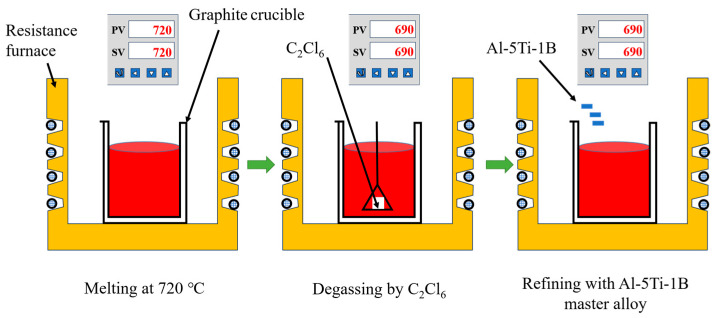
Schematic diagram of the Al-Si-Mg alloy preparation process.

The Al-Si-Mg aluminum alloy used in this study was prepared using purity aluminum (4 N), Si (7 N), and Al-Mg master alloy (Al-20 wt.% Mg). The alloy was processed according to the procedure depicted in Figure 1. The aluminum alloy was heated to 720 °C for melting and held at 690 °C for 30 min. It was then degassed by adding C_2_Cl_6_ and held for 15 min. Al-5Ti-B master alloy (1 wt.%) was added to refine the grains, and the melt was held for 15 min before starting the semi-solid slurry preparation. The chemical compositions of the GISS and SEED slurries were determined separately, as presented in Table 1. 

### 2.2. Slurry Preparation and Die-Casting

**Figure 2:**
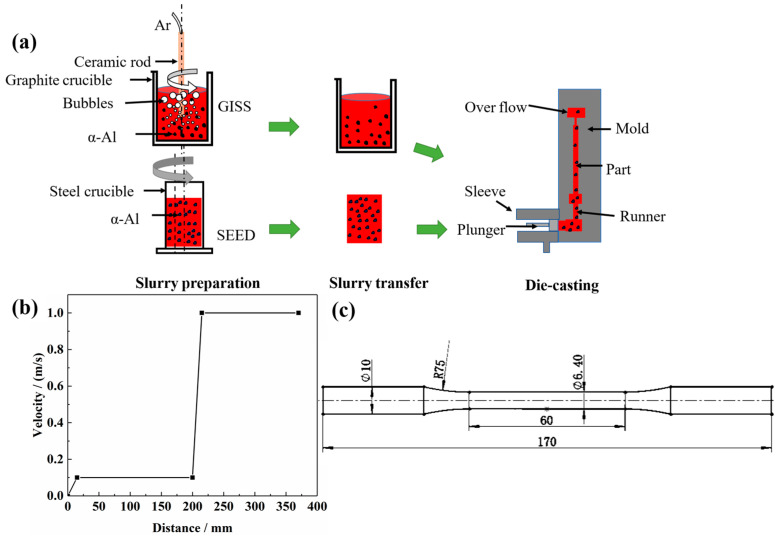
(**a**) Slurry preparation and die-casting process; (**b**) Injection speed curve; (**c**) Dimensions of the test bar.

As illustrated in Figure 2a, the slurry preparation route of GISS involved cooling the melt by injecting high-purity argon gas at a flow rate of 0.35 m^3^/h through a ceramic rod. The ceramic rod was immersed in the melt at a temperature of 625 °C for 4 min, with a revolution speed of 90 r/min and a rotation speed of 120 r/min. After the stirring, the slurry was held for 85 s. After completing the slurry preparation, it was transferred to a shoot sleeve of the HPDC machine for the production of parts with varying injection speeds, as depicted in Figure 2b. 

During the SEED-HPDC process, the melt was poured into a steel crucible at a temperature of 625 °C. It was then rotated eccentrically at 150 r/min for 100 s. The resulting semi-solid slurry was transferred to the shot sleeve to obtain castings using the same die-casting process parameters as the GISS-HPDC process. 

The details of the T6 heat treatment were as follows: the test bars were subjected to a solution treatment at a temperature of 540 °C for 0.5 h in a furnace (CGF1200-25) with a temperature fluctuation of ±1 °C. After the solution treatment, they were quenched in 60 °C water. Subsequently, the quenched bars were transferred to a furnace (DHG-9076A) with a temperature fluctuation of ±1 °C for aging at 170 °C for 6 h.

### 2.3. Microstructure Characterization and Mechanical Properties Tests

Metallographic samples of slurry and parts were ground, polished, and then etched using a 0.5% HF solution. The microstructure was observed using a Nikon metallographic microscope (Nikon LV100ND, Tokyo, Japan). Phase identification and fracture morphology were analyzed using a JEOL-7610F scanning electron microscope (SEM). The phase analysis of the samples was conducted by X-ray diffraction (Rigaku, Ultima IV, Tokyo, Japan) with Cu-K_α_ radiation at a speed of 10° per minute between 10° and 90°. The solid fraction, average grain size (D), and average shape factor (F) were analyzed using ImageJ software (Version 1.53t, https://imagej.nih.gov/ij/index.html). The solid fraction (Fs) was estimated by measuring the volume fraction of the large light gray α-Al grains, which were preserved during quenching from a semi-solid state. The detailed procedure for the measurement is given in [17]. During the quantitative image analysis, at least 300 grains for the same sample were measured. The equations used for the analysis are as follows (Equations (1) and (2)):

(1) $$D=\frac{\Sigma_{i=1}^{N}\sqrt{4A_{i}∕\pi }}{N}$$

(2) $$F=\frac{\Sigma_{i=1}^{N}\sqrt{4A_{i}}}{P_{i}^{2}}$$

where $A_{i}$ is the area grains, $P_{i}$ is the perimeter of grains, and N and i are the number of grains.

Figure 2c illustrates the dimensions of the test bars. Tensile tests were conducted using the Zwick-Z250 tensile machine (ZwickRoell GmbH & Co., Ulm, Germany), with a tensile speed of 1 mm/min (GB/T 228.1-2010). At least two samples in the same conditions were tested for repeatability. The Vickers hardness tests were carried out using an MH-3 micro-hardness tester, and the average of 8 data points was measured. A load of 200 gf was applied and maintained for 15 s on polished samples (GB/T 4340.1-2009).

## 3. Results

### 3.1. Microstructure of Al-Si-Mg Alloy in Various Slurry Preparation Routes

**Figure 3:**
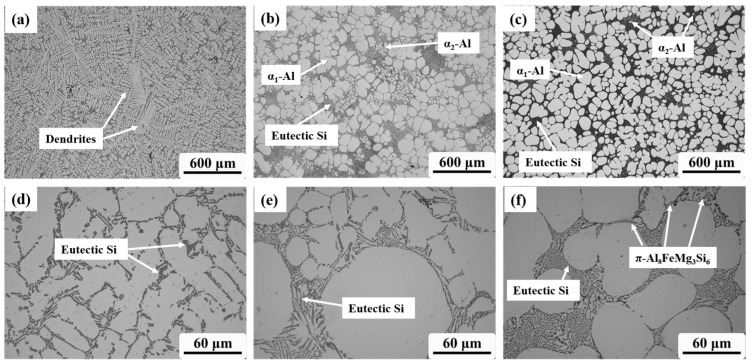
Microstructure obtained by different preparation methods: (**a**,**d**) taking sample at 660 °C, (**b**,**e**) GISS method, (**c**,**f**) SEED method, and all three samples were water quenched.

**Figure 4:**
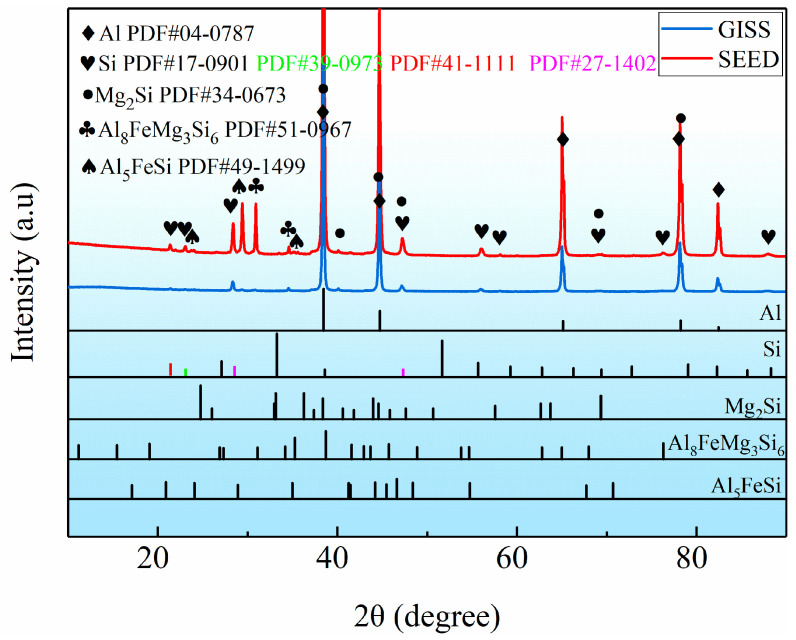
XRD patterns of semi-solid Al-Si-Mg alloys prepared by GISS and SEED.

Figure 3 compares the microstructure of the Al-Si-Mg aluminum alloys obtained through different slurry preparation methods. In Figure 3a, a conventional liquid solidified microstructure is depicted, exhibiting a typical α-Al dendrite morphology with eutectic Si distributed around the dendrites. On the other hand, Figure 3b,c show the semi-solid microstructure prepared by the GISS and SEED methods, respectively. These microstructures are characterized by near-spherical grains distributed in the liquid phase. Table 2 presents statistical results for the two slurry microstructures, including the solid volume fraction, average grain size, and average shape factor of α_1_-Al grains. The results indicate that the semi-solid slurry prepared by GISS exhibits characteristics of a 55% solid fraction, an average grain size of 105 μm, and an average shape factor of 0.85. On the other hand, the SEED method results in a slurry with a 75% solid fraction, an average grain size of 95 μm, and an average shape factor of 0.60. Additionally, the α_2_-Al grains and eutectic Si phase in the slurries prepared by these two methods are also distributed around α_1_-Al. The α_2_-Al grains are formed by the solidification of the residual melt during the die-casting process. The grain size of α_2_-Al grains is much smaller compared to α_1_-Al grains. The number of α_2_-Al grains in GISS slurry is much higher than that in SEED slurry, as shown in Figure 3b,c. Moreover, Figure 3d reveals that the conventional HPDC sample contains a larger block-like eutectic Si phase in addition to the plate-like eutectic Si phase. From Figure 3e,f, it can be observed that most eutectic Si phases in GISS slurry are in the form of long fibers. In contrast, in SEED slurry, the smaller liquid pools result in a smaller space for the distribution of the eutectic Si phases, leading to a denser distribution and a smaller size of eutectic Si phases [18]. Based on the XRD results of the GISS and SEED samples shown in Figure 4, it can be inferred that the π-Fe phase and the β-Fe phase are present in the SEED semi-solid slurry. However, the β-Fe phase is not clearly indicated in Figure 3f due to the excessive density of the eutectic Si phase.

The morphology of α_1_-Al grains prepared by the GISS method is predominantly spherical, while in SEED, α_1_-Al grains exhibit a more complex and irregular morphology, appearing near spherical or rosette-shaped. Although both semi-solid slurry preparation methods operate under a nucleation-controlled mechanism, there are certain differences in the slurry produced using the two preparation methods. The use of a graphite crucible in GISS results in a lower cooling rate, more uniform temperature, and solute distribution in the melt, leading to the formation of more spherical α_1_-Al grains. Additionally, GISS involves a longer holding time, which contributes to larger α_1_-Al grains. Tang [19] observed a significant decrease in the average grain size with an increasing cooling rate, while Gu [17] discovered that the grains grew and even developed dendritic structures through the Oswald ripening mechanism with prolonged resting time.

The spheroidization mechanism of the GISS process involves the combined stirring effect of a stirring rod and argon. This results in a more uniform temperature and solute distribution in the melt, leading to nearly equal growth rates in all directions. This uniform growth is beneficial for the spherical growth of grains [20]. The formation of primary spherical α_1_-Al grains in SEED is influenced by various parameters such as pouring temperature and eccentric rotation speed and time. The combined effects of high grain density, slow cooling, and rotational convection reduce temperature and solute gradients at the solidification interface, allowing grains to grow in a spherical morphology. Qu [21] developed a Phase-Field-Lattice-Boltzmann model to explain the evolution mechanism of spherical grains in SEED slurry. This model helps to reveal the key control factors for the formation of spherical grains and predict microstructure evolution in other processes.

### 3.2. Microstructure of the Rheo-HPDC Samples

**Figure 5:**
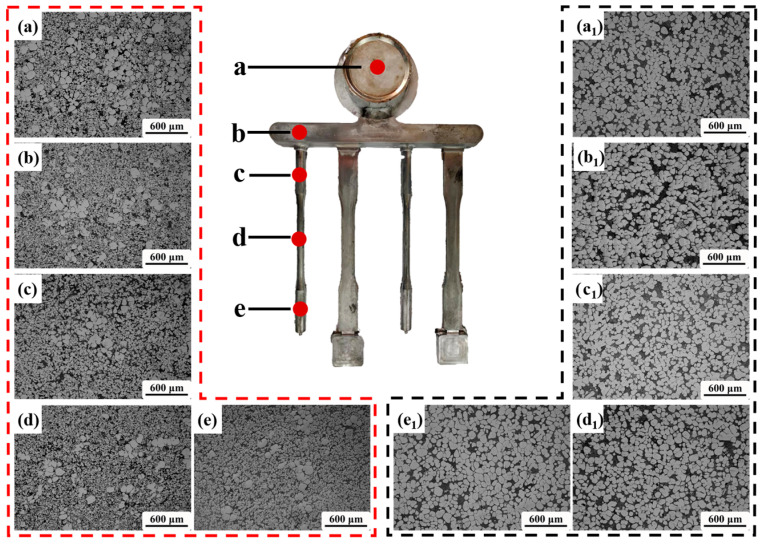
Microstructure at different positions of the part along the die-casting filling direction, (**a**) location ‘a’; (**b**) location ‘b’; (**c**) location ‘c’; (**d**) location ‘d’; (**e**) location ‘e’ produced byGISS-HPDC; and (**a_1_**) location ‘a’; (**b_1_**) location ‘b’ (**c_1_**) location ‘c’; (**d_1_**) location ‘d’; (**e_1_**) location (**e**) produced by SEED-HPDC.

In order to investigate the flow behavior and segregation during the rheo-HPDC process, the microstructure changes in parts of GISS-HPDC and SEED-HPDC were analyzed. Figure 5 displays the microstructures at different positions of the part. The observation positions are marked with red points labeled as ‘a–e’. Figure 5a–e shows the microstructure of the GISS-HPDC at different positions, including the biscuit, runner, and various areas of the test bar. From the figures, it can be observed that the proportion of α_1_-Al grains gradually decreases along the filling direction of the slurry, and a large number of small α_2_-Al grains surround the spherical α_1_-Al grains. On the other hand, Figure 5(a_1_–e_1_) illustrates the SEED-HPDC microstructures at corresponding positions. It can be seen that the microstructure is evenly distributed in all positions, with no significant difference in the proportion of α_1_-Al grains. The microstructure of the part is similar to that of the slurry. 

**Figure 6:**
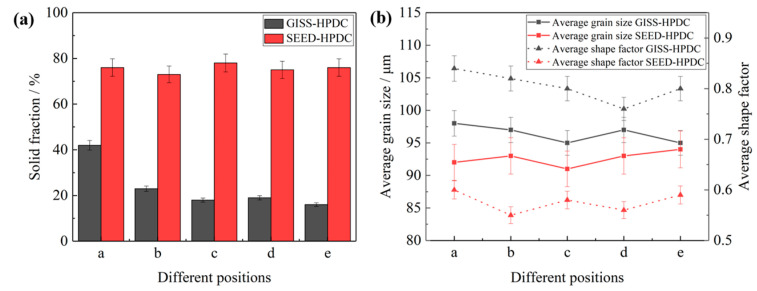
Statistical results of α_1_-Al in GISS-HPDC and SEED-HPDC samples: (**a**) solid fraction; (**b**) average grain size and average shape factor.

Figure 6 presents a comparison of the statistical results for the solid volume fraction, average grain size, and average shape factor of α_1_-Al grains in different positions (shown in Figure 5) prepared by using the GISS and SEED methods. The figure shows that in the GISS-HPDC process, the solid volume fraction decreases from 40% to around 20%, exhibiting a significant variation from the biscuit to the end of the test bar. The segregation of α_1_-Al grains during the filling process is observed to be severe. However, the average grain size and average shape factor remain relatively stable, ranging from 95–100 μm and 0.75–0.85, respectively. On the other hand, in the SEED-HPDC process, the solid fraction is around 75–80%, the average grain size ranges between 90 and 95 μm, and the average shape factor is between 0.55 and 0.60. Compared to the GISS-HPDC statistics, the most noticeable difference in SEED-HPDC is that the solid volume fraction remains essentially constant, and the microstructure demonstrates excellent uniformity.

**Figure 7:**
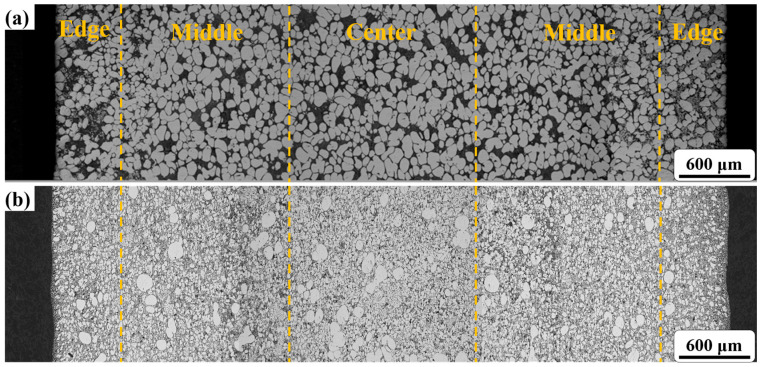
Cross-sectional microstructure of test bars from center to edge: (**a**) GISS-HPDC; (**b**) SEED-HPDC.

**Figure 8:**
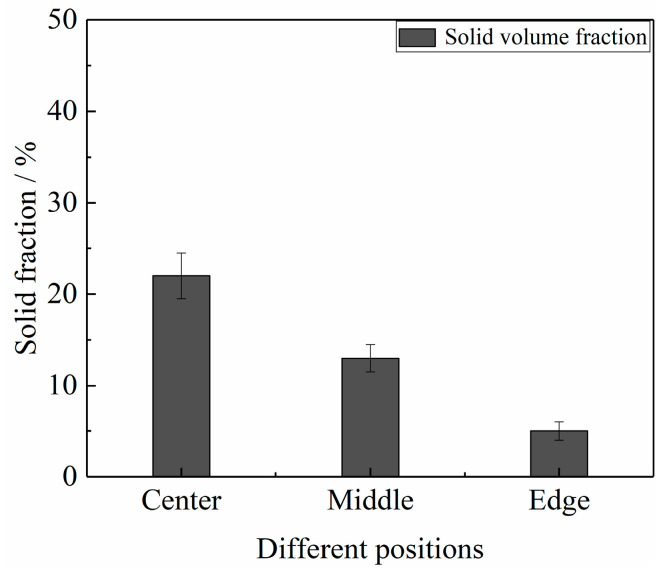
Statistical results of α_1_-Al at the cross-section of GISS-HPDC test bar.

Figure 7 shows the cross-sectional microstructure of the test bar, specifically at position ‘c’ in Figure 6. The microstructure of the SEED-HPDC sample in the cross-section (Figure 7a) is found to be similar to its microstructure along the filling direction, exhibiting minimal surface segregation. However, the GISS-HPDC sample exhibits different characteristics. The center region of the GISS-HPDC sample consists of spherical α_1_-Al grains and a large number of α_2_-Al grains. Moving towards the middle region, the number of α_1_-Al and α_2_-Al grains decreases, accompanied by the presence of eutectic silicon-rich areas. The edge region, which is in contact with the mold wall, primarily contains α_2_-Al grains with only a few α_1_-Al grains. In addition, Figure 8 shows the statistical results for the α_1_-Al grains of the GISS-HPDC test bar. These results indicate a gradual decrease in the solid fraction from 22% at the center to 5% at the edge region. Due to the rapid cooling rate and high liquid fraction in rheo-HPDC, the grains did not undergo plastic deformation, thereby maintaining a microstructure similar to that of the semi-solid slurry.

### 3.3. Mechanical Properties of A356 Aluminum by Different Die-Casting Methods

**Figure 9:**
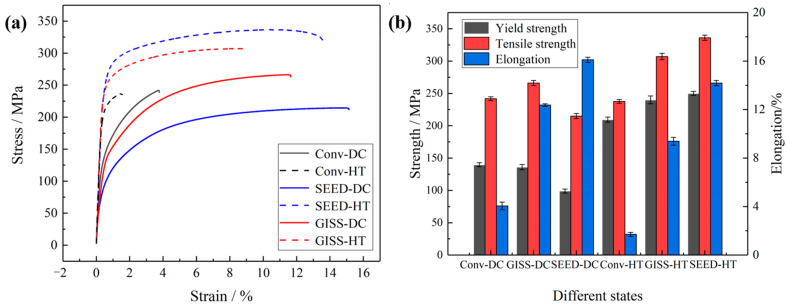
Tensile properties of SEED-HPDC and GISS-HPDC parts: (**a**) stress−strain curve; (**b**) strength and elongation values of the parts.

Table 3 and Figure 9 show the mechanical properties of the test bars produced by conventional HPDC, heat-treated conventional HPDC (Conventional-HT), SEED-HPDC, and GISS-HPDC in various heat treatment states. The yield strength, tensile strength, and elongation of the conventional HPDC samples are 139 MPa, 241 Mpa, and 3.7%, respectively. GISS-HPDC samples exhibit higher yield and tensile strengths of 136 MPa and 266 MPa, which are 37.4% and 23.7% higher than those of SEED-HPDC samples. Both heat-treated GISS-HPDC (GISS-HT) and SEED-HPDC (SEED-HT) samples show increased strengths, but experience reduced elongation. The SEED-HT samples demonstrate yield and tensile strengths of 249 MPa and 336 MPa, which are 3.8% and 9.4% higher than those of GISS-HT. Table 4 also compares the mechanical properties of the alloys with similar chemical compositions under different forming processes and different heat treatment processes.

**Figure 10:**
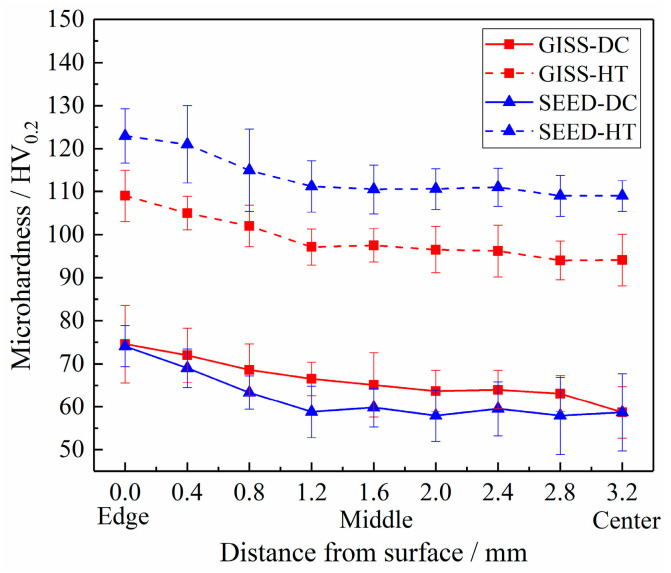
Evolution of microhardness from edge to center of the studied parts in the different states.

Figure 10 reveals the evolution of microhardness in the cross-sectional direction, ranging from the edge (0 mm) to the center (3.2 mm) of the samples. It is observed that as the distance from the surface decreases, the microhardness increases. At the center region, the microhardness values for the GISS-DC, SEED-DC, GISS-HT, and SEED-HT samples are 59 HV_0.2_, 59 HV_0.2_, 94 HV_0.2_, and 109 HV_0.2_, respectively. Similarly, at the edge region, the values are 75 HV_0.2_, 74 HV_0.2_, 109 HV_0.2_, and 123 HV_0.2_ for the respective samples. The GISS-HT and SEED-HT samples both exhibit an increase in microhardness, which is consistent with their performance in terms of strength. Furthermore, the difference in microhardness between the SEED-DC and GISS-DC samples is not significant, indicating that the hardness test is not affected by defects like porosity and oxidation. The higher microhardness obtained in the edge region of all samples can be attributed to the presence of a relatively higher eutectic phase enriched in alloying elements [27].

## 4. Discussion

### 4.1. Segregation during Die-Casting

**Figure 11:**
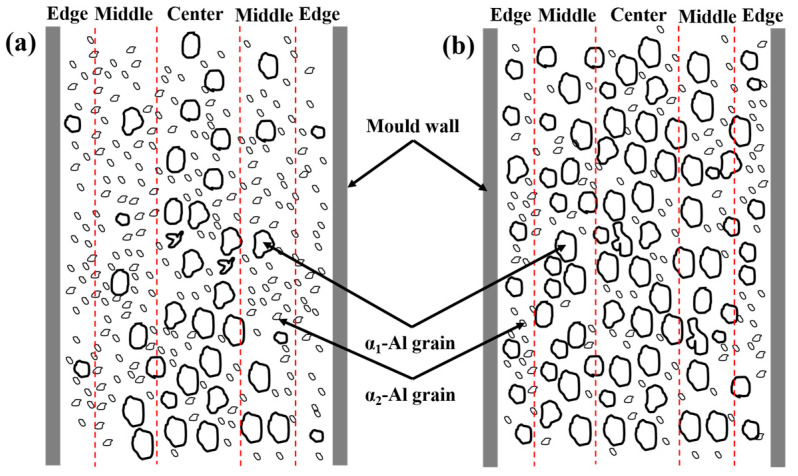
Segregation model in the rheo-HPDC samples: (**a**) GISS-HPDC; (**b**) SEED-HPDC.

The microstructure distribution in the rheo-HPDC process varies depending on the solid fraction of the semi-solid slurry, as shown in Figure 5 and Figure 7. The flow characteristics of semi-solid slurry can be categorized into liquid-like slurry and solid-like slurry [28]. The semi-solid slurry prepared by GISS is considered a liquid-like slurry with a low solid fraction and flow behavior similar to a liquid. In this type of semi-solid slurry, there is minimal interaction between α_1_-Al grains. Figure 11a illustrates the segregation model of the GISS-HPDC process. As the filling pressure decreases during the filling sequence, α_1_-Al grains in the flowing slurry forefront lack sufficient driving force to overcome the viscosity of the residual liquid and fill the designated positions. The liquid phase with better flowability is squeezed to the slurry forefront and edge region, leading to a decrease in the volume fraction of α_1_-Al grains along the filling direction. The edge region, which is in contact with the mold wall, experiences a high cooling rate, causing the liquid phase in this region to solidify rapidly and form a large number of α_2_-Al grains. The remaining slurry with α_1_-Al grains rapidly solidifies from the edge to the center, resulting in an increase in the volume fraction of α_1_-Al grains with increasing distance from the mold wall [29,30]. Li [31] prepared a semi-solid slurry with a solid fraction of 25% by the self-inoculation method and die-casted the products, and also observed a decrease in the solid fraction along the filling direction. 

The slurry prepared by SEED belongs to the solid-like slurry. The slurry looks solid in its static state and becomes thinner when it is sheared. In Figure 5 and Figure 7, the plastic formation of grains during the SEED-HPDC process was not found. In addition, most grains were surrounded by a thin liquid layer. This indicates the grains have a slight connection. The filling of the slurry is driven by the liquid and retains a quasi-uniform microstructure [32]. The segregation model of the SEED-HPDC is shown in Figure 11b. It is normal to find slight segregation in the edge region contact with the mold wall.

Segregation is a common and unavoidable defect in conventional HPDC and rheo-HPDC processes, which is influenced by elemental contents, structural design, process parameters, and the solid fraction. Li [30] conducted a study on the segregation of rheo-HPDC samples of 6061 aluminum alloy with varying Si contents. The study found that liquid-phase segregation improved with increasing Si contents. The pouring temperature plays a crucial role in the size and morphology of α_1_-Al grains, which, in turn, affects their flow behavior and segregation during the die-casting process. Zhang [33] controlled the pouring temperature to obtain grains with different morphologies (dendrites and globular grains) and found that the dendritic structure led to severe segregation in the die-casting parts. Qi [34] investigated the segregation phenomenon of AC46000 castings at different wall thickness locations, where different wall thicknesses represented varying cooling rates. The study revealed that higher cooling rates lead to severe segregation of the primary phase. Jiao [35] and Gourlay [36] investigated the segregation band on the circular cross-section of the test bar during the HPDC process and found the position of the segregation band moved to the mold wall with increasing mold temperature. As the solid fraction increases, the segregation band varies from poorly defined multiple bands to well-defined single bands disappearing.

### 4.2. Difference in Mechanical Properties between GISS-HPDC and SEED-HPDC

Qi [7] produced A356 aluminum alloy parts through the conventional HPDC process. The yield strength, tensile strength, and elongation of the parts were measured to be 151 MPa, 228 MPa, and 4.3%, respectively. These mechanical property results are consistent with those obtained in our study. However, due to the limitations of the conventional HPDC process, subsequent heat treatment was not conducted, which hindered the further enhancement of the performance of the A356 alloy. In Figure 9, the tensile strengths of the samples produced by GISS-DC and conventional-DC are higher than those of SEED-DC. It is evident from Figure 3 and Figure 5 that the microstructure of conventional HPDC and GISS-DC contains a significant number of fine α_2_-Al grains, resulting in an overall reduction in the grain size of the product. This contributes to the improvement of their mechanical properties. The strength of the samples is enhanced after T6 heat treatment. This attributes to solution treatment and artificial aging treatment [37,38]. Solution treatment is conducted at a high temperature to dissolute Mg-rich phases and promote the homogenized distribution of elements in the Al matrix. It can also spheroidize eutectic Si. The contribution of artificial aging to strength depends on the precipitation of the Mg-Si strengthening phase. Coherent or semi-coherent Mg-Si precipitates a strengthening effect that results from the Orowan mechanism. The tensile strength of SEED-HT parts is higher than that of GISS-HT ones. The reasons for the differences in mechanical properties can be discussed in terms of defects and the microstructure of the test bars.

**Figure 12:**
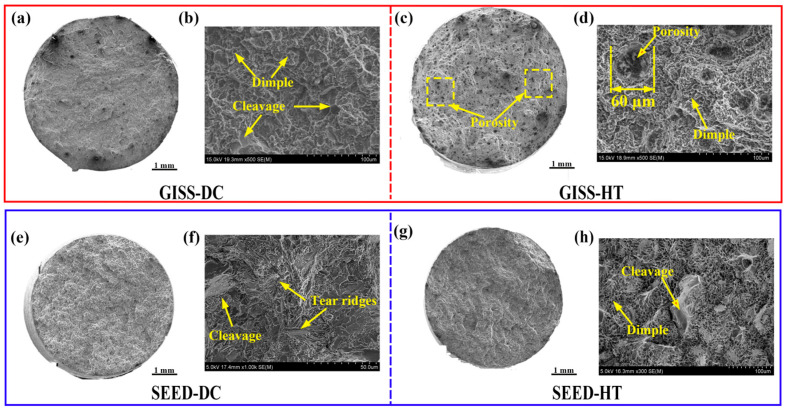
SEM fractographs of GISS-HPDC and SEED-HPDC samples in different states: (**a**,**b**) GISS-DC; (**c**,**d**) GISS-HT; (**e**,**f**)SEED-DC; (**g**,**h**) SEED-HT.

Figure 12 shows the fracture morphology of GISS-HPDC and SEED-HPDC test samples in different states. The GISS-DC samples exhibit characteristics of ductile fracture, including dimples and minor cleavage. On the other hand, the SEED-DC samples display quasi-cleavage features, with some cleavage and tearing ridges present in their fracture morphology. In the case of GISS-HT samples, the fracture morphology reveals a large number of porosities throughout the cross-section. This occurrence of porosities could be attributed to the low solid fraction during the filling process, which results in air entrapment. Upon reheating the parts to the solution temperature, the high temperature causes the expansion of entrapped gas, leading to the formation of porosities. The mechanism can be explained by Equation (3) [3]:

(3) $$\frac{P_{1}V_{1}}{T_{1}}=\frac{P_{2}V_{2}}{T_{2}}$$

where P, V, T represent the pressure, volume, and temperature of the entrapped gas, respectively. 

When the sample is reheated from $T_{1}$ to a higher temperature $T_{2}$ during the solution treatment, the pressure will increase from $P_{1}$ to a higher pressure $P_{2}$. If the pressure $P_{2}$ exceeds the deformation resistance $F_{R}$ of the alloy at that temperature, the alloy will undergo plastic deformation and form blister defects. As the volume of porosity increases, the pressure $P_{2}$ will decrease. Additionally, $F_{R}$ will decrease as the component temperature increases. Once the pressure $P_{2}$ is smaller than $F_{R}$, the growth of porosity will stop. 

The porosity level is a critical factor that significantly affects the mechanical properties of die-casting parts [2]. During tensile tests, the presence of porosity defects can lead to stress concentration, crack initiation, and propagation, ultimately reducing the mechanical properties of the part [39]. Notably, the SEED-HT samples exhibit minimal porosities on the cross-section of the fracture, indicating that the use of a high-solid fraction semi-solid slurry results in parts with a denser structure and fewer defects. Consequently, the SEED-HT samples showcase superior strength and elongation compared to the GISS-HT ones.

**Figure 13:**
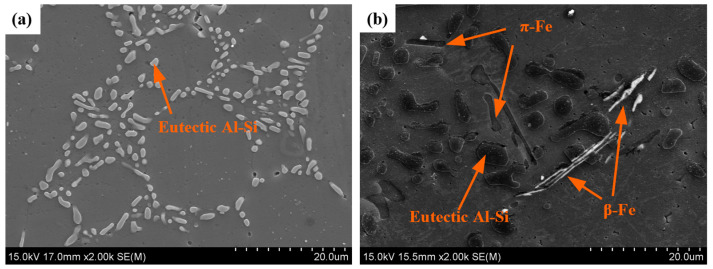
Morphology of the second phase after heat treatment: (**a**) GISS-HT; (**b**) SEED-HT.

Figure 13 displays the microstructure of the GISS-HT and SEED-HT samples. Fine near-globular eutectic Si particles and α-Al were observed in the GISS-HT samples. However, the SEED-HT samples consist of near-globular eutectic Si particles, needle-shape β-Fe, and rod-like π-Fe, which were confirmed by EDS analysis as presented in Table 5.

The transformation of fiber-shaped eutectic Si into near-globular eutectic Si can reduce the lacerating effect of eutectic Si on the matrix and improve the mechanical properties. Chen [40] investigated the precipitation behavior of Si-containing dispersoids in Al-7Si-Mg aluminum alloys during the solution process and found that part of the Si atoms precipitated in the form of Si-containing dispersions. The presence of these Si-containing dispersions increases the microhardness of the alloys. Chen [24] investigated the effect of different Mg contents on the mechanical properties of the Al-7Si-Mg alloy and suggested that with increasing Mg contents, the density of the Mg-Si strengthening phases increased. This enhances the interaction between the precipitated phase and dislocations during deformation, leading to an increase in the yield strength and tensile strength but a decrease in the elongation of the alloy. However, excessive Mg contents lead to an increase in the number and size of iron-rich intermetallic compounds (π-Al_8_FeMg_3_Si_6_), which have a detrimental effect on mechanical properties [41]. 

The presence of Fe-intermetallic compounds in aluminum alloys can be detrimental as they can cause damage to the aluminum matrix, create stress concentration, and reduce the mechanical properties of the aluminum alloy [42]. Furthermore, the β-Fe phase precipitates before the aluminum grains in the solidification process, resulting in the formation of coarse and long needle-shaped β-Fe phases. These phases hinder the flow of the melt, leading to reduced fluidity and the potential for defects like shrinkage and porosities [43]. However, in the Al-Si alloy, the element Fe can have positive effects when present below the critical content. In this case, the strength, hardness, and high-temperature properties will increase with increasing Fe contents [44]. Although Fe-intermetallic compounds were found in the SEED-HPDC samples, the mechanical properties and elongation of the samples remained high after heat treatment. Future research will focus on investigating the Fe content and the morphology of Fe-intermetallic compounds to further enhance the mechanical properties of the alloys.

## 5. Conclusions

The tensile test bars of the Al-Si-Mg aluminum alloy were successfully manufactured by GISS-HPDC and SEED-HPDC. The GISS and SEED methods were employed to prepare semi-solid slurries. After investigating and analyzing the microstructure, segregation, and mechanical properties of the samples, the following conclusions were drawn:

1. The dendritic microstructure in conventional HPDC samples can be effectively replaced by spherical primary α-Al grains through GISS and SEED methods combined with rheo-HPDC. The slurry prepared by SEED, which has a lower liquid fraction (~25 ± 4%), exhibits a smaller grain size (95 ± 4.2 μm) and denser eutectic Si phases compared to the slurry prepared by GISS. However, the shape factor of the α-Al grains prepared by SEED (~0.60) is lower as compared to that of GISS (~0.8).
2. The phenomenon of surface liquid segregation is observed in both SEED-HPDC and GISS-HPDC samples. Furthermore, in the GISS-HPDC samples, there is a gradual increase in the liquid fraction along the filling direction. However, the SEED-HPDC samples with a low liquid fraction (<25%) in the rheo-HPDC process with proper process parameters help to minimize the occurrence of segregation. As a result, these slurries with low liquid fractions enable the production of parts with a more uniform microstructure.
3. Both heat-treated GISS-HPDC and SEED-HPDC samples exhibit noticeably higher strength and elongation when compared to conventional HPDC samples. Moreover, the SEED-HT samples demonstrate superior mechanical properties in comparison to the GISS-HT samples, owing to their denser structure and reduced porosity. The yield strength, tensile strength, and elongation of the SEED-HT samples are measured at 249 MPa, 336 Mpa, and 13.7%, respectively.
4. Although good mechanical properties were obtained for the GISS-HPDC samples after heat treatment, further investigation is needed to minimize porosities during this process. Additionally, it is important to study the improvement of the shape factor for primary α-Al grains in SEED slurries and its impact on flow behavior.

## Figures and Tables

**Table 1:** Chemical composition of Al-Si-Mg aluminum alloy (wt.%).

	Si	Cu	Mg	Fe	Ti	Al
GISS	6.840	0.026	0.492	0.027	0.037	Bal
SEED	6.45	0.098	0.41	0.13	0.05	Bal

**Table 2:** Comparison of α1-Al grains statistics in GISS and SEED slurry microstructure.

	Solid Fraction	Average Grain Size/μm	Average Shape Factor
GISS	55 ± 4.8%	105 ± 3.0	0.85
SEED	75 ± 4.0%	95 ± 4.2	0.60

**Table 3:** Tensile properties of SEED-HPDC and GISS-HPDC parts.

	Yield Strength/MPa	Tensile Strength/MPa	Elongation/%
Conventional-DC	139 ± 3	241 ± 3	3.7 ± 0.3
Conventional-HT	210 ± 7	266 ± 8	1.6 ± 0.3
SEED-DC	99 ± 4	215 ± 4	15.1 ± 0.1
GISS-DC	136 ± 3	266 ± 4	11.6 ± 0.2
SEED-HT	249 ± 6	336 ± 5	13.7 ± 0.3
GISS-HT	240 ± 3	307 ± 4	8.8 ± 0.2

**Table 4:** Comparison of mechanical properties for several Al-Si-based alloys.

Alloys	Forming Process	YS−UTS−EL MPa−MPa−%	Solution	Aging	Ref.
Al-7Si-0.3Mg-0.1Ti-0.1Fe	Low-pressure casting	210−285−14.0	540 °C−4 h	155 °C−3 h	[22]
Al-7Si-0.4Mg-0.2Ti-0.13Fe-0.1Cu	Semi-solid squeeze casting	201−283−8	540 °C−3 h	160 °C−9 h	[23]
Al-7Si-0.6Mg-0.13Fe-0.13Ti	Sand casting	301−312−0.6	550 °C−2 h	180 °C−22 h	[24]
Al-8.2Si-0.53Fe-0.46Mg-0.15Cu-0.01Sr	HPDC	129.5−208.7−2.2	-	-	[25]
Al-8.2Si-0.53Fe-0.46Mg-0.15Cu-0.01Sr	Rheo-HPDC	279.9−358.2−12.5	535 °C−1 h	175 °C−2.2 h	[26]

**Table 5:** EDS quantitative results of β-Al5FeSi and π-Al8FeMg3Si6 (at%).

Fe Phase	Al	Si	Fe	Mg
β-Al_5_FeSi	72.04	13.43	14.23	0.30
π-Al_8_FeMg_3_Si_6_	43.60	32.10	5.30	19.00

## Data Availability

The data that support the findings of this study are available from the corresponding author upon reasonable request.
